# Supplementary material for: Structure and functional implications of WYL domain-containing bacterial DNA damage response regulator PafBC
Source: Nat Commun. 2019 Oct 11;10:4653. doi: 10.1038/s41467-019-12567-x (PMC6789036; doi:10.1038/s41467-019-12567-x)
Supplement: Supplementary file 1 — Supplementary Information [file 41467_2019_12567_MOESM1_ESM.pdf]

# Supplementary Information

**Structure and functional implications of WYL domain-containing bacterial DNA damage response regulator PafBC**

Andreas U. Müller et al.

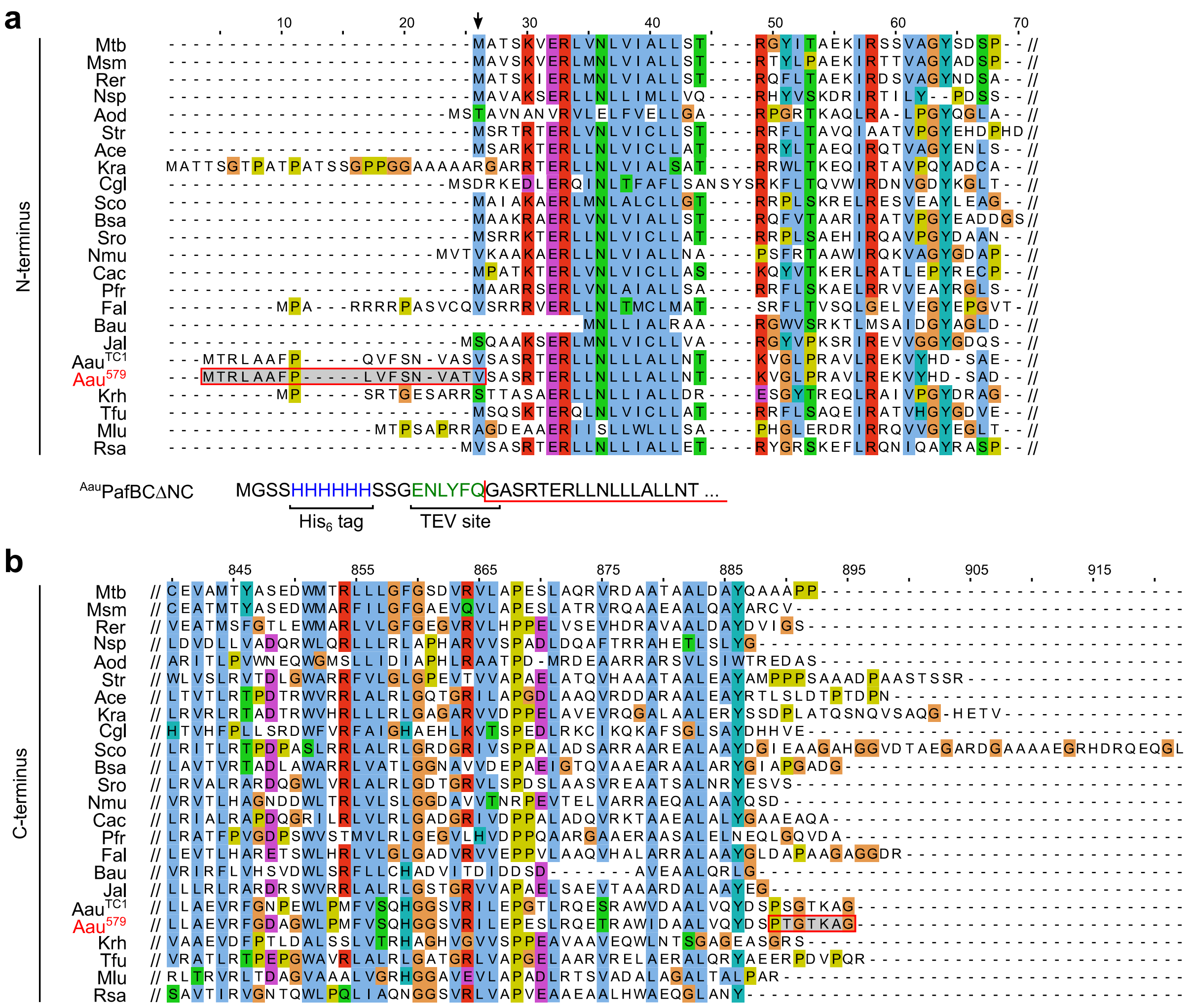

**Supplementary Figure 1: Sequence alignment of the N- and C-terminal regions of PafBC orthologs.** (a) The N-terminus of ${}^{Aau}$PafBC from strain 579 (red, Aau$^{579}$) is likely a misannotation in the database, because most other PafB proteins contain a conserved initiator methionine at alignment position 26 (arrow at the top) and the valine in *A. aurescens* may be due to usage of an alternative start codon, which is common among *Actinobacteria*. For crystallization experiments, the additional 17 residues of Aau$^{579}$ from the N-terminus were removed (including the theoretical initiator methionine, red box with gray background), and the first serine was replaced with glycine to generate a TEV protease cleavage site (see also experimental procedures). The N-terminal sequence of the construct expressed and used for crystallization (${}^{Aau}$PafBCΔNC) is shown below. The red line marks the N-terminus of the purified protein. (b) The C-terminal residues of Aau$^{579}$ are not conserved among other actinobacterial PafC proteins, and thus, 7 C-terminal residues are not contained in ${}^{Aau}$PafBCΔNC, which was used for crystallization. Residues are colored according to the ClustalX color scheme. Numbers above the sequences refer to the alignment position numbers.

Mtb = *Mycobacterium tuberculosis* (P9WIM1, P9WIL9), Msm = *Mycobacterium smegmatis* (I7G3U5, A0QZ41), Rer = *Rhodococcus erythropolis* (C0ZZU3, C0ZZU2), Nsp = *Nocardioides sp.* (A1SK18, A1SK19), Aod = *Actinomyces odontolyticus* (A7BCC5, A7BCC6), Str = *Salinispora tropica* (A4X749, A4X750), Ace = *Acidothermus cellulolyticus* (A0LU62, A0LU63), Kra = *Kineococcus radiotolerans* (A6W976, A6W977), Cgl = *Corynebacterium glutamicum* (Q8NQE2, Q8NQE3), Sco = *Streptomyces coelicolor* (Q9RJ64, Q9RJ65), Bsa = *Blastococcus saxobsidens* (H6RJ02, H6RJ01), Sro = *Streptosporangium roseum* (D2ATU2,

D2ATU1), Nmu = *Nakamurella multipartita* (C8XAP4, C8XAP3), Cac = *Catenulispora acidiphila* (C7PVW0, C7PVW1), Pfr = *Propionibacterium freudenreichii* (A0A160VN40, A0A161KHT8), Fal = *Frankia alni* (Q0RLT0, Q0RLS9), Bau = *Brevibacterium aurantiacum* (A0A1D7W444, A0A1D7W495), Jal = *Jiangella alkaliphila* (A0A1H2KTF9, A0A1H2KTV3), $Aau^{TC1}$ = *Arthrobacter aurescens* strain TC1 (A1R6R2), Krh = *Kocuria rhizophila* (B2GIN6), Tfu = *Thermobifida fusca* (Q47P13), Mlu = *Micrococcus luteus* (C5CBV3), Rsa = *Renibacterium salmoninarum* (A9WSH6)

UniProt accession numbers are given in parentheses. Refers to Figure 1 and experimental procedures.

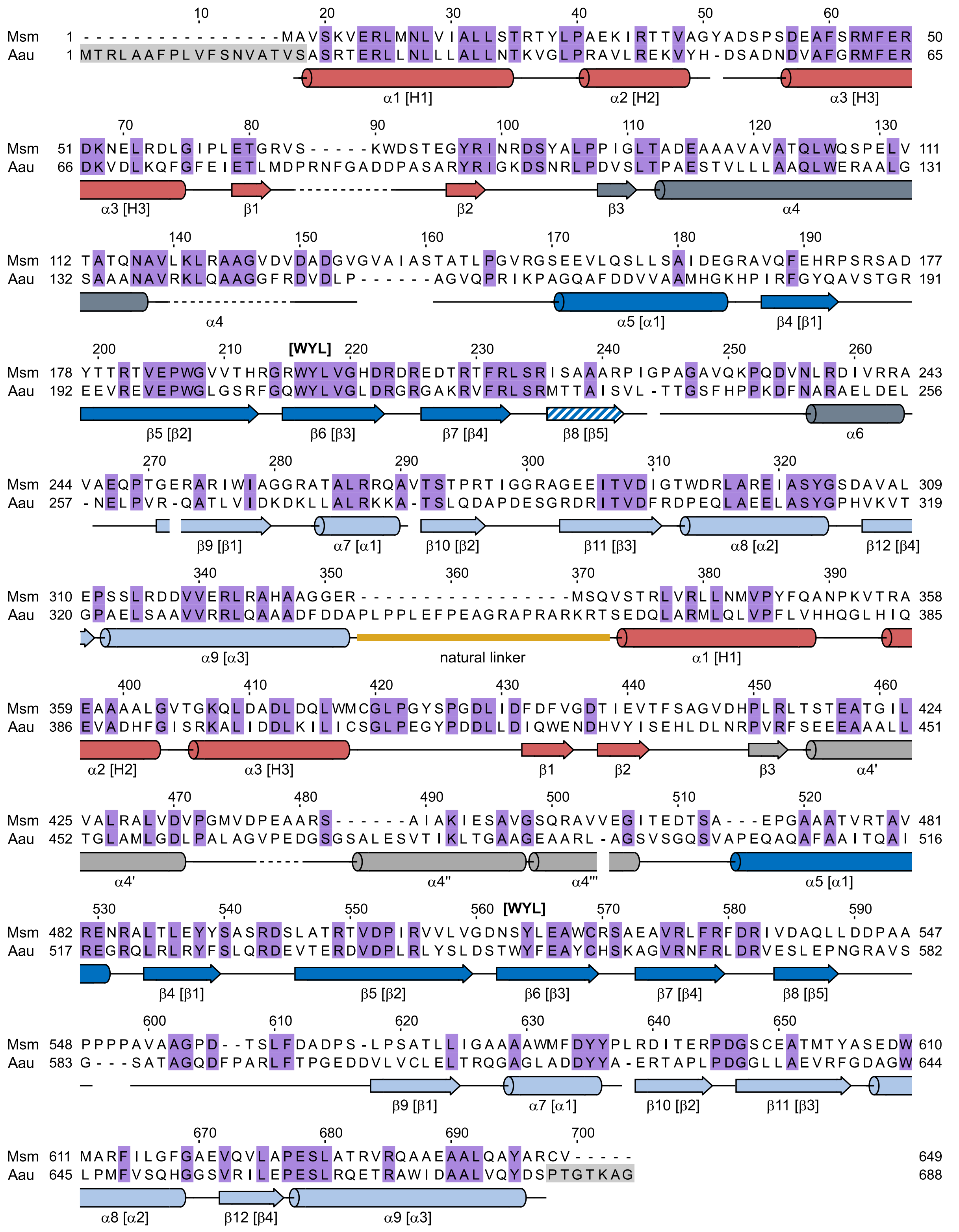


**Supplementary Figure 2: Secondary structure mapping of the $^{Aau}$PafBCΔNC crystal structure to the protein sequences of PafBC from *Arthrobacter aurescenes* and *Mycobacterium smegmatis*.** Equivalent secondary structure elements in PafB and PafC were numbered the same. Internal numbering and/or designations of secondary structure elements for individual domains are given in brackets. The WYL motif is indicated in bold letters above the alignment. The last β-strand of WYL-B (β5) was inferred from WYL-C (hatched coloring). A dashed main chain indicates gaps in the model. Identical residues in both organisms are colored in violet. Numbers above the sequences refer to the

alignment position numbers. Gray residues were not part of the protein used for crystallization. Refers to Figure 1.

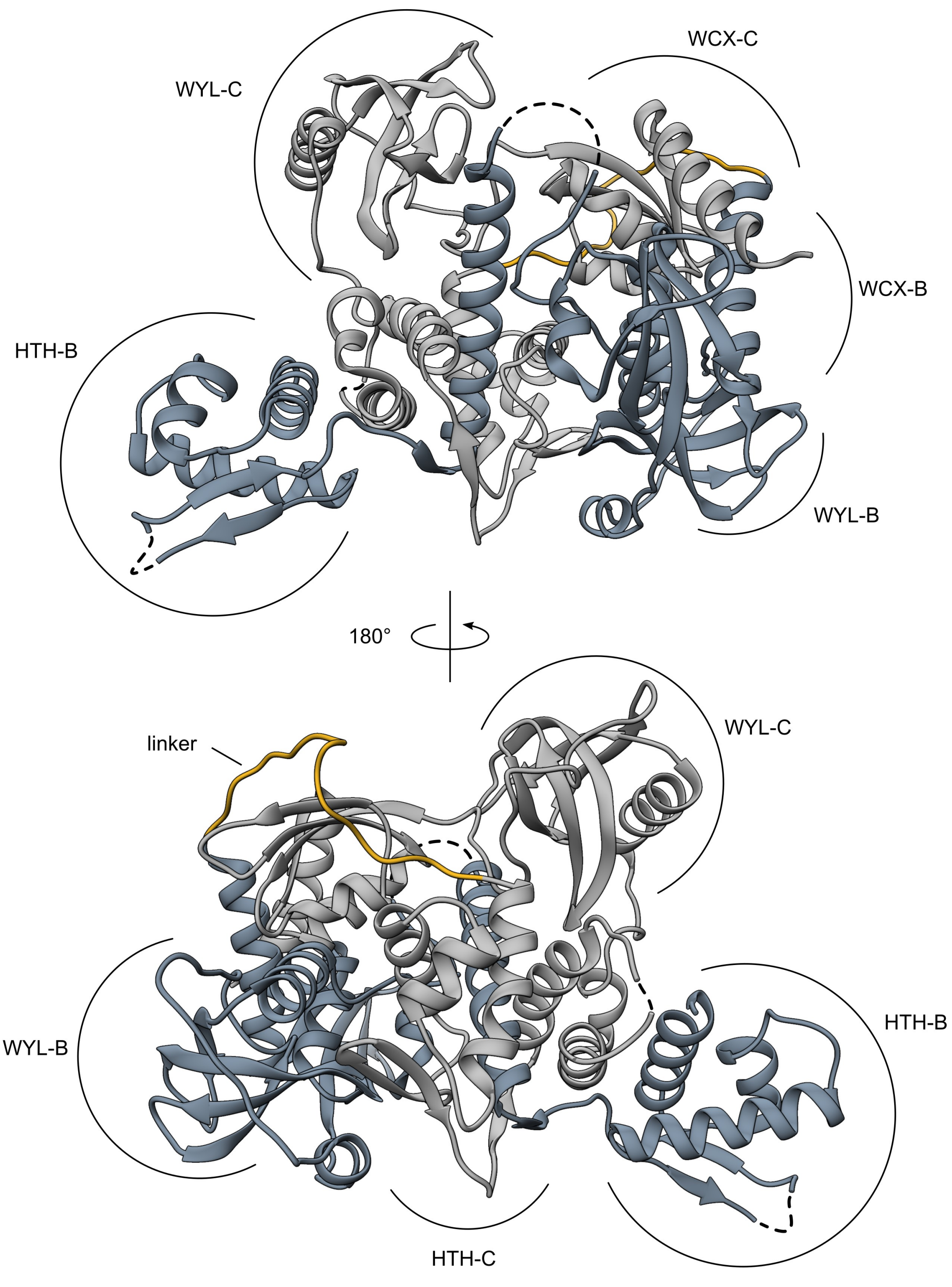


**Supplementary Figure 3: Extended overview of the $^{Aau}$PafBCΔNC crystal structure.** The PafB part is colored in dark gray and the PafC part in light gray. The natural linker present in $^{Aau}$PafBC is colored in yellow. Refers to Figure 1.

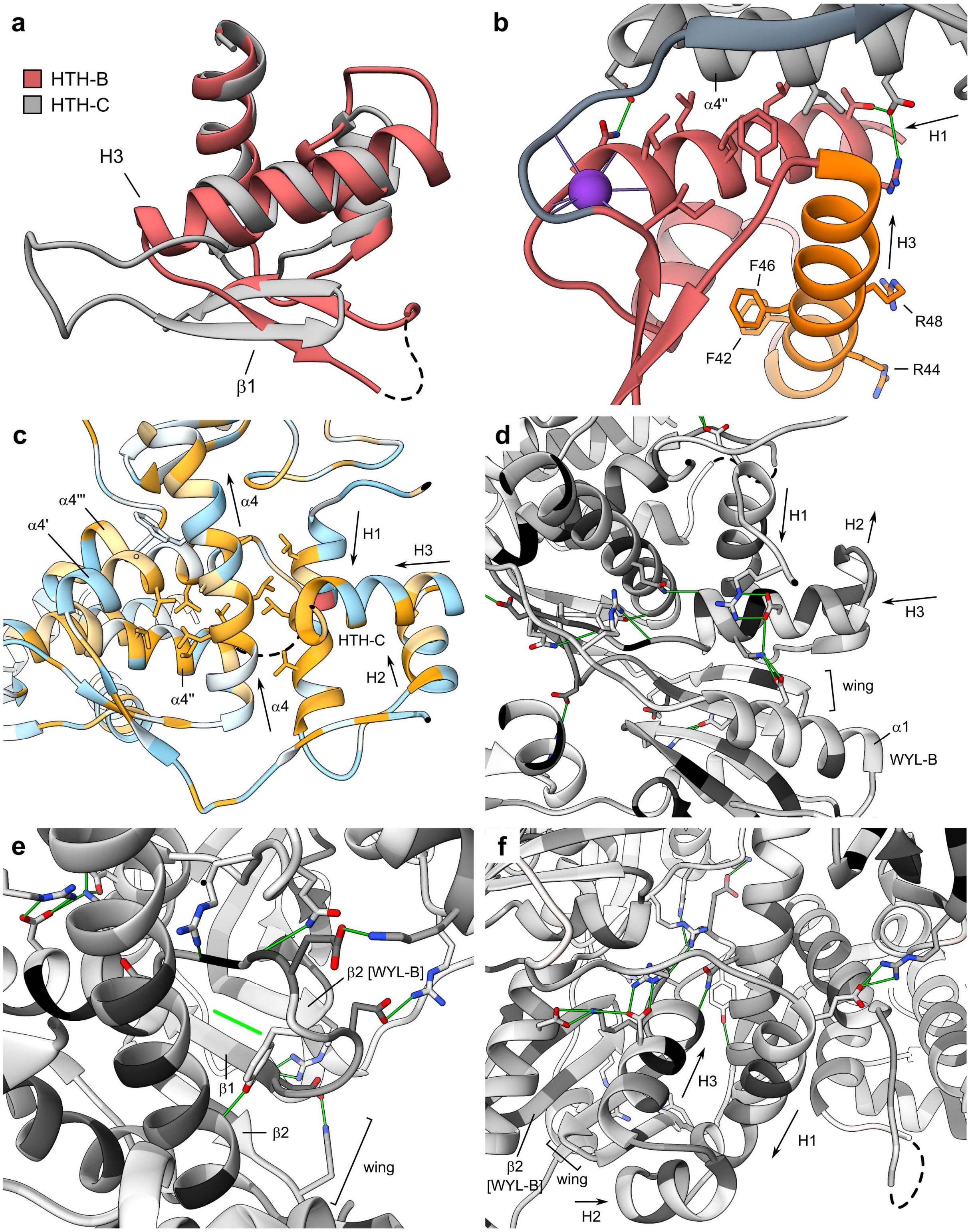


**Supplementary Figure 4: HTH domains of $^{Aau}$PafBCΔNC interact with other parts of the molecule to a very different degree.** (a) The HTH domain of PafC (HTH-C, gray) has a shorter H3 and a longer loop connecting to the β1 strand of the wing compared to the HTH domain of PafB (HTH-B, red), while the β1/β2 loop is much shorter in HTH-C. (b) H1 of HTH-B interacts with helix α4'' (part of the helix bundle in PafC) almost exclusively through hydrophobic interactions (residues in stick representation). The recognition helix of HTH-B (H3; orange) contains two highly conserved phenylalanines involved in forming the hydrophobic core of the domain and two highly conserved, exposed arginines that could make specific base contacts in the DNA-bound form of PafBC. A potassium ion (lilac) is caged in

between the main chain of H1 and the wing. (c) The central helix α4 (part of PafB) makes hydrophobic interactions with H1 of PafC and α4'' of PafC. Residues were colored based on their hydrophobicity according to the Kyte-Doolittle scale from orange (hydrophobic) to cyan (polar). Selenomethionines are colored in light red. Leucine, valine and phenylalanine are shown in stick representation. (d-f) Additionally, HTH-C interacts with the protein core through a variety of hydrogen bonds, mostly involving the main chain and non-conserved residues. The hydrogen bonds stabilizing the extended β-sheet formed by the HTH-C wing and WYL-B are collectively depicted as single green line in panel e. Conservation is colored from black (conserved) to white (no conservation) based on the BLOSUM-62 matrix. Dashed lines depict gaps in the model. Refers to Figure 2.

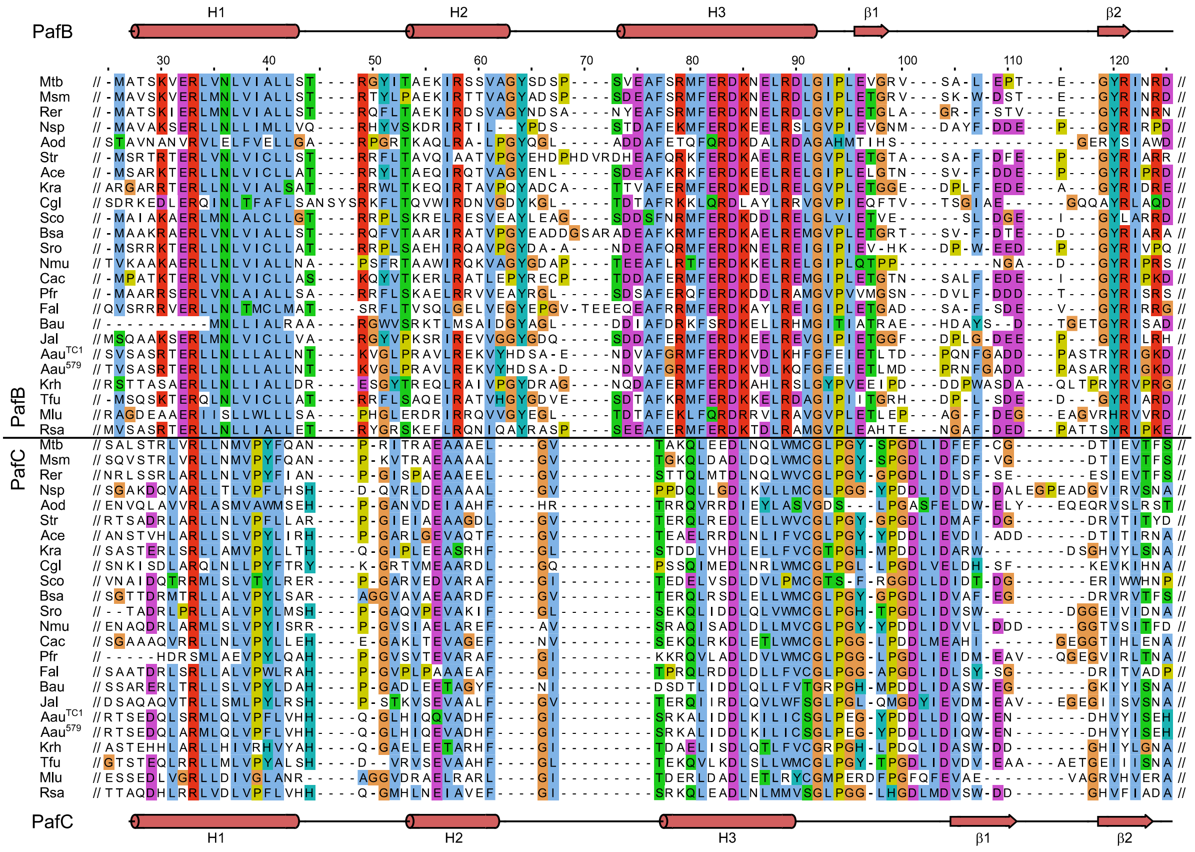

**Supplementary Figure 5: Sequence alignment of the HTH domains of PafBC across the actinobacterial phylum.** The β1 strand of HTH-C occupies a position different from the β1 strand in HTH-B. Secondary structure elements of the $^{Aau}$PafBCΔNC crystal structure are shown for HTH-B (above) and HTH-C (below). Residues are colored according to the ClustalX color scheme. Numbers above the sequences refer to the alignment position numbers.

Mtb = *Mycobacterium tuberculosis* (P9WIM1, P9WIL9), Msm = *Mycobacterium smegmatis* (I7G3U5, A0QZ41), Rer = *Rhodococcus erythropolis* (C0ZZU3, C0ZZU2), Nsp = *Nocardioides sp.* (A1SK18, A1SK19), Aod = *Actinomyces odontolyticus* (A7BCC5, A7BCC6), Str = *Salinispora tropica* (A4X749, A4X750), Ace = *Acidothermus cellulolyticus* (A0LU62, A0LU63), Kra = *Kineococcus radiotolerans* (A6W976, A6W977), Cgl = *Corynebacterium glutamicum* (Q8NQE2, Q8NQE3), Sco = *Streptomyces coelicolor* (Q9RJ64, Q9RJ65), Bsa = *Blastococcus saxobsidens* (H6RJ02, H6RJ01), Sro = *Streptosporangium roseum* (D2ATU2, D2ATU1), Nmu = *Nakamurella multipartita* (C8XAP4, C8XAP3), Cac = *Catenulispora acidiphila* (C7PVW0, C7PVW1), Pfr = *Propionibacterium freudenreichii* (A0A160VN40, A0A161KHT8), Fal = *Frankia alni* (Q0RLT0, Q0RLS9), Bau = *Brevibacterium aurantiacum* (A0A1D7W444, A0A1D7W495), Jal = *Jiangella alkaliphila* (A0A1H2KTF9, A0A1H2KTV3), Aau$^{TC1}$ = *Arthrobacter aurescens* strain TC1 (A1R6R2), Krh = *Kocuria rhizophila* (B2GIN6), Tfu = *Thermobifida fusca* (Q47P13), Mlu = *Micrococcus luteus* (C5CBV3), Rsa = *Renibacterium salmoninarum* (A9WSH6)

UniProt accession numbers are given in parentheses. Refers to Figure 2.

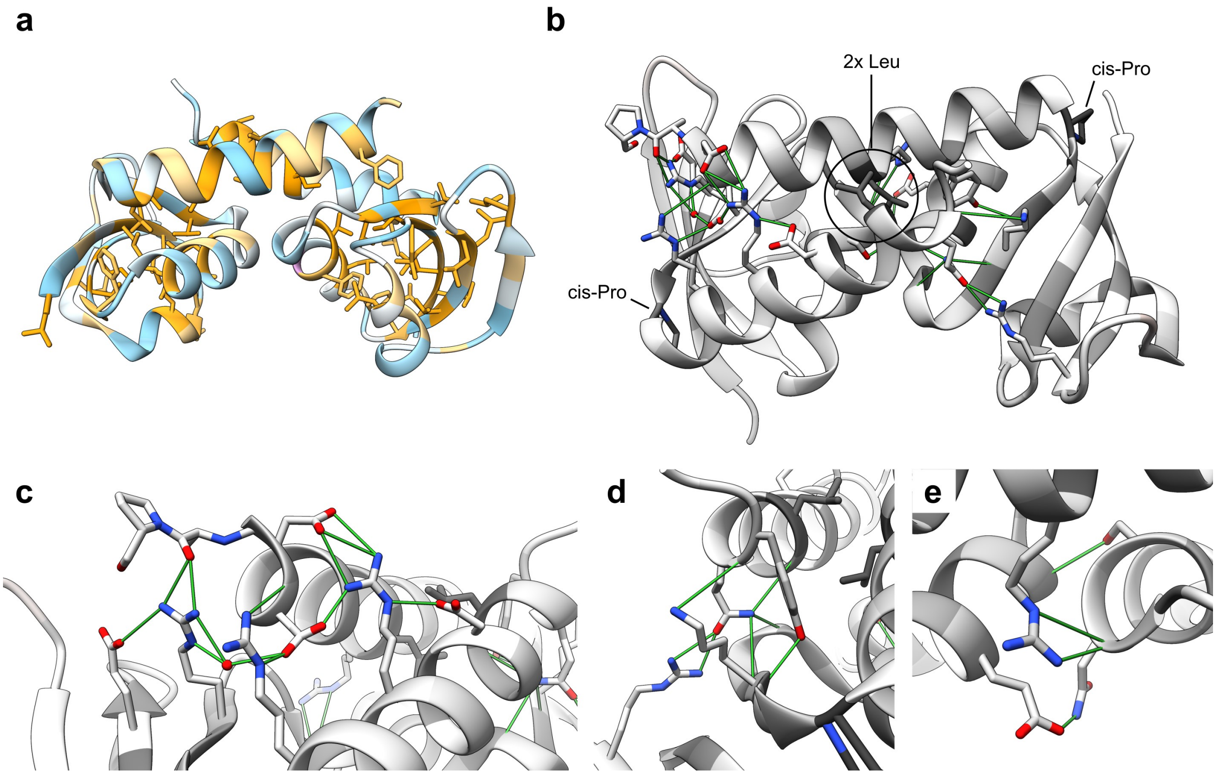


**Supplementary Figure 6: The C-terminal extension (WCX) domains of PafBC form an interaction module.** (a) Hydrophobicity coloring reveals a hydrophobic core in the ferredoxin-like fold of each WCX domain. Hydrophobicity is colored from orange (hydrophobic) to cyan (polar) according to the Kyte-Doolittle scale. Leu, Val, Phe are shown in stick representation. (b-e) The WCX domains of PafBC contain a network of hydrogen bonds and salt bridges (green) around two highly conserved leucines located in a small hydrophobic island at the C-terminal helix. The residue conservation is colored from black (conserved) to white (no conservation) based on the BLOSUM-62 matrix. Refers to Figure 3.

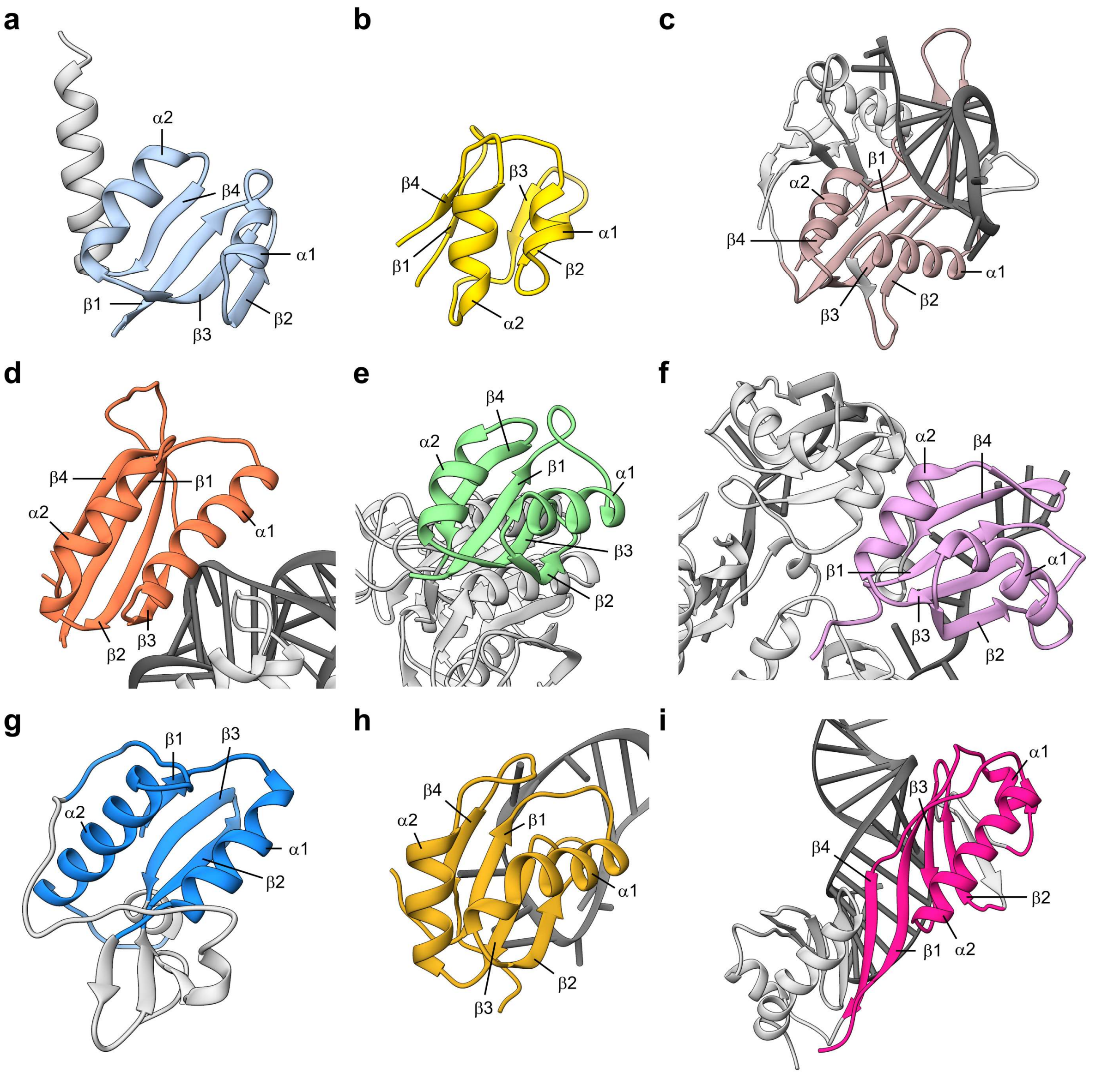


**Supplementary Figure 7: Structural comparison of selected proteins containing a ferredoxin-like fold.** (a) *Arthrobacter aurescens* PafBC WCX-C domain (b) *Thermotoga maritima* ferredoxin (PDB 1VJW) (c) *Thermus thermophilus* Cse3 bound to RNA substrate (PDB 2Y8W) (d) Ribosomal protein S6 from *T. thermophilus* bound to rRNA (PDB 1G1X) (e) *Salmonella typhimurium* subtilisin (PDB 1SBP) (f) Human hnRNP A1 protein UP1 bound to an RNA ligand (PDB 6DCL) (g) T4 phage translational regulator protein RegA (PDB 1REG) (h) *Drosophila melanogaster* protein U1A/SNF bound to RNA (PDB 6F4H) (i) Yeast TATA-binding protein (PDB 1YTB). Only the ferredoxin-like fold is shown in color, while other parts are shown in light gray. Secondary structure elements in addition to the classical βαββαβ topology of the ferredoxin-like fold are colored in light gray as well. Nucleic acid ligands are colored in dark gray. Refers to Figure 3.

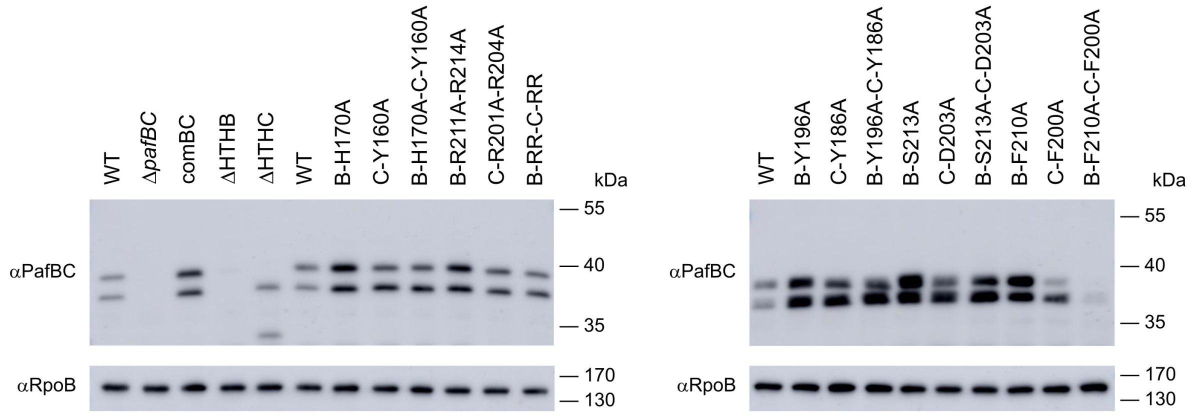


**Supplementary Figure 8: Expression levels of *Mycobacterium smegmatis* PafBC variants in the complemented strains.** PafBC variants were expressed from an integrative plasmid in the *M. smegmatis* Δ*pafBC* strain and the expression levels were compared to the knockout (Δ*pafBC*) and wild-type (WT) strains carrying the empty plasmid. RpoB served as loading control. A representative immunoblot of four individual experiments is shown. Refers to Figure 5.

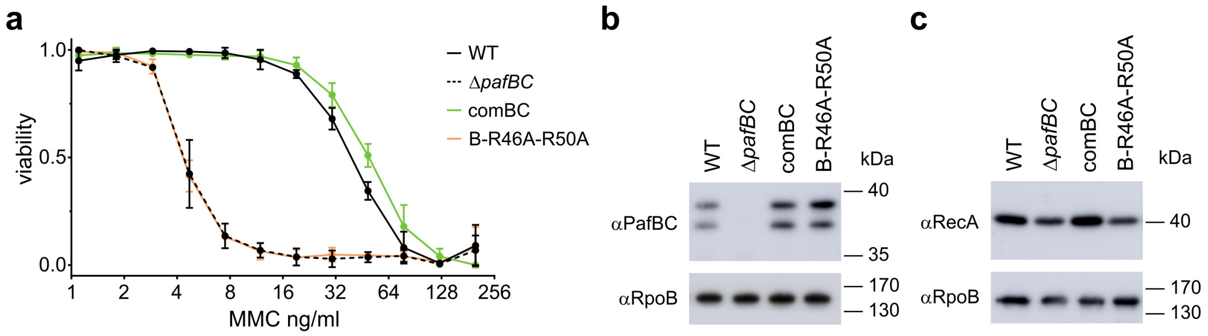


**Supplementary Figure 9: Mutation of two highly conserved arginines in the HTH domain PafB (HTH-B) disrupts PafBC function.** (a) Complementation of the *M. smegmatis* Δ*pafBC* strain with a PafBC variant carrying the R46A and R50A mutations in HTH-B (B-R46A-R50A) does not restore viability to wild type level measured by the resazurin assay with mitomycin C (MMC). Data points are given as the mean of three individual experiments and error bars represent the standard deviation of the mean. (b) The HTH-B arginine double mutant expression levels were compared to the knockout (Δ*pafBC*) and wild type (WT) strains carrying the empty plasmid and a complement strain expressing wild type PafBC (comBC). RpoB served as loading control. A representative immunoblot of two individual experiments is shown. (c) RecA is not induced in the HTH-B arginine double mutant under mitomycin C stress. Strains were grown to OD600 of 1.0 and exposed to 80 ng/ml MMC for 4 h before immunoblotting RecA. RpoB served as loading control. Refers to Figure 5.

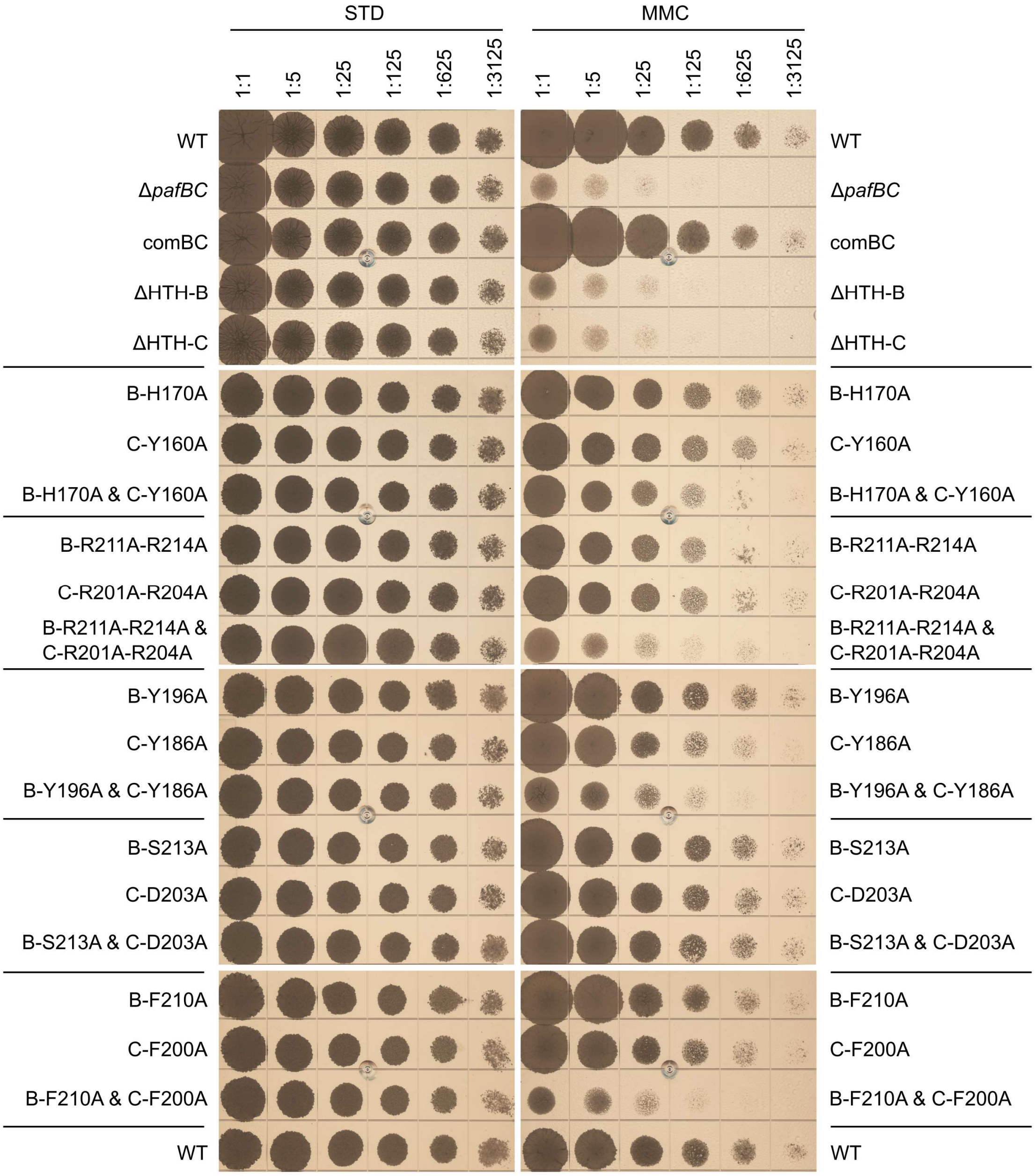


**Supplementary Figure 10: Viability testing of PafBC mutants by spotting on agar plates confirms the results of the resazurin assay.** *M. smegmatis* Δ*pafBC* strains complemented with PafBC variants were exposed to mitomycin C (MMC; 80 ng/ml) for 4 h before preparing the indicated dilutions and spotting onto agar plates. Refers to Figure 5.

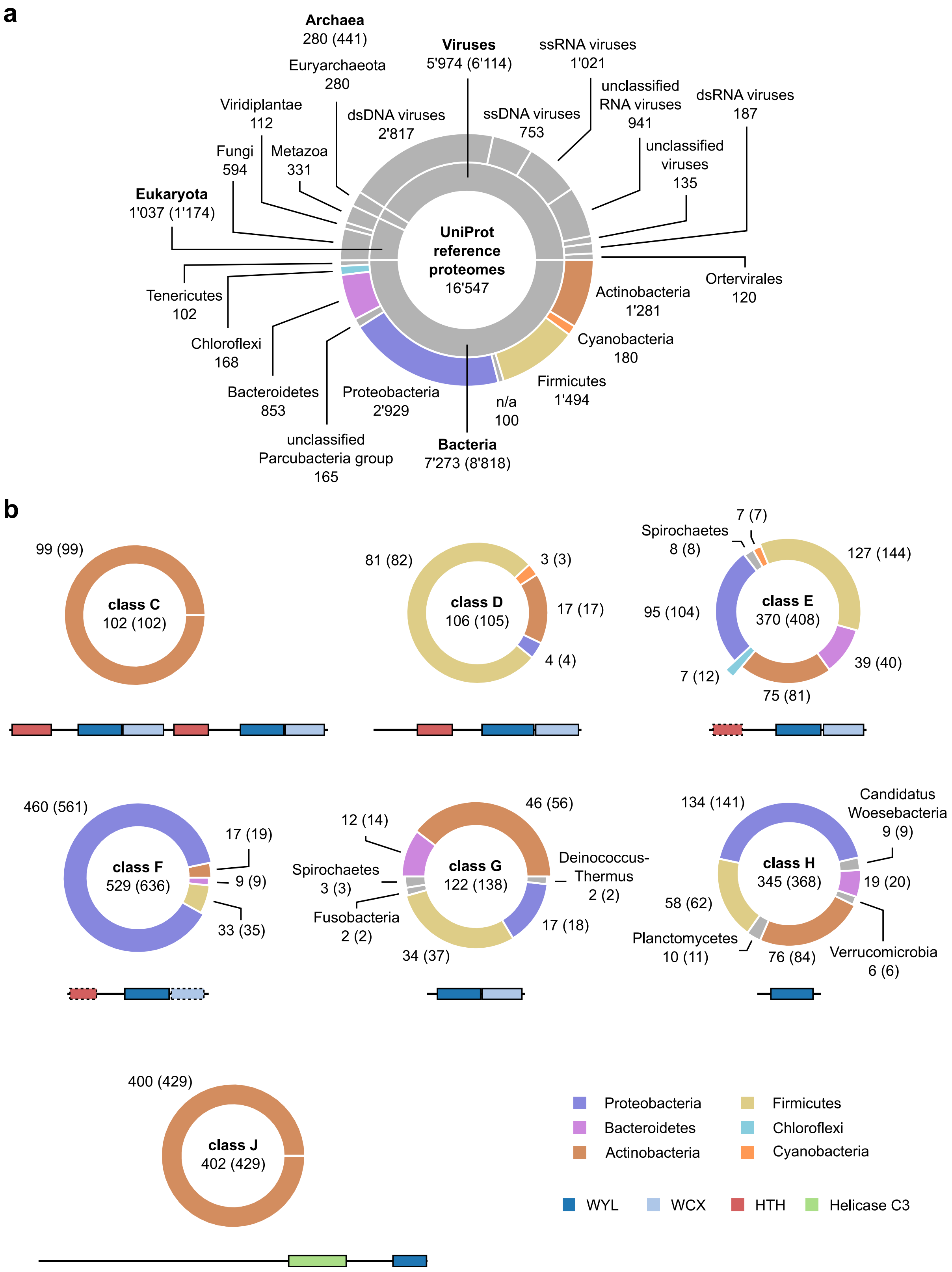


**Supplementary Figure 11: Taxonomic distributions of the UniProt reference proteomes and domain architecture classes of proteins containing the WYL domain.** (a) Taxonomic distribution within UniProt reference proteomes. The six largest taxonomic groups of the bacteria superkingdom were color-coded. Groups smaller than 5% of the total number of reference proteomes were omitted for better visualization. (b) Taxonomic distributions among domain architecture classes show that class C, D, F,

and J are largely specific to one of the bacterial superkingdoms. Groups smaller than 1.5% of the total number of reference proteomes were omitted for better visualization. Refers to Figure 6.

**Supplementary Table 1: Oligonucleotide primers used in this study.** Overhangs for Gibson assembly are shown in lowercase letters.

| Primer name | Sequence (5' -> 3') | Purpose |
|---|---|---|
| aaubc-fw | gagaatctttattttcagGGCAGTGGAACGCGCCTTGCTGCTTTCC | Amplification of *pafBC* from *Arthrobacter aurescens* genomic DNA |
| aaubc-rv | ctcgagtgcggccgcCTAGCCAGCCTTGGTGCCCG | See aaubc-fw |
| temp_lin-fw | GCGGCCGCACTCGAG | Linearization of temporary vector |
| temp_lin-rv | ctgaaaataaagattctcGCCGCTGCTGTGATGATG | See temp_lin-rv |
| aaubcdNC-fw | gaacctgtacttccagggcGCATCCCGCACCGAACG | Amplification of truncated $^{Aau}$PafBC |
| aaubcdNC-rv | gctcgagtgcggccgcttaTGAGTCGTACTGCACCAAAG | See aaubcdNC-fw |
| pET28a_linHT-rv | GCCCTGGAAGTACAGGTTCTC | Linearization of customized pET28a (already encodes His-TEV site) |
| pET28a_lin-fw | GCGGCCGCACTCGAG | See pET28a_linHT-rv (identical sequence to temp_lin-fw) |
| pMyNT-ori-fw | TTGAATACCAGCCAGACGAGACGG | Exchange of the pMyNT replicative origin with integrative L5 cassette |
| pMyNT-ori-rv | ACGAGCCACACAGCACCCGAAC | |
| L5cassette1-fw | cgggtgctgtgtggctcgtCCATACACCGGTGTACGATC | |
| L5cassette1-rv | CGAAGATCTCAGCGGCGAC | |
| L5cassette2-fw | cgccgctgagatcttcgTGCACTACCGGATCGCGGCAT | |
| L5cassette2-rv | cgtctggctggtattcaaTCCCCAATTGTTGCTAGCTGG | |
| p0pafA-fw | gaagtgacgcggtctcaGTAGAAGTGCTGGACCGACAC | Cloning of complementation vector wild-type *pafBC* |
| p0pafA-rv | gactttggagaccgccacAACATCGAGCTTACGGGTTGC | |
| pafBC-fw | TGGCGGTCTCCAAAGTCG | |
| pafBC-rv | cgtcgacatcgataagctttTCATACGCAACGGGCATAGG | |
| pMyNTint-fw | AAGCTTATCGATGTCGACGTAGTTAAC | |
| pMyNTint-rv | TGAGACCGCGTCACTTCTTTATC | |
| deltaHTHB-fw | CGGGATTCCTACGCGCTG | Deletion of PafB HTH domain |
| deltaHTHB-rv | GACCGCCACAACATCGAGC | |
| deltaHTHC-fw | GTGACGTTCTCCGCAGGC | Deletion of PafC HTH domain |
| deltaHTHC-rv | CTGACTCATCGTTCCCCTCC | |
| B-H170A-fw | CGGCCGTCGCGCAG | Mutational screen |
| B-H170A-rv | GGCCTCGAACTGCACGGCCCG | |
| C-Y160A-fw | TACTCGGCCTCGCGCG | Mutational screen |
| C-Y160A-rv | GGCCTCCAGGGTCAGCGCCC | |
| B-R211A-R214A-fw | ATCAGCGCCGCGGCCC | Mutational screen |
| B-R211A-R214A-rv | GGCTGAAAGCGCGAAGGTTCTGGTGTCCTC | |
| C-R201A-R204A-fw | ATCGTCGACGCCCAACTGC | Mutational screen |
| C-R201A-R204A-rv | GGCGTCGAAAGCGAACAGCCGCACGGCCTC | |
| B-Y196A-fw | CTCGTCGGCCACGACCG | Mutational screen |
| B-Y196A-rv | GGCCCAGCGGCCGCGATGG | |
| C-Y186A-fw | CTGGAGGCGTGGTGCC | Mutational screen |
| C-Y186A-rv | GGCGCTGTTGTCGCCCACGAG | |
| B-S213A-fw | CGCATCAGCGCCGCG | Mutational screen |
| B-S213A-rv | TGCAAGCCGGAAGGTTCTGGTG | |
| C-D203A-fw | CGCATCGTCGACGCCC | Mutational screen |
| C-D203A-rv | GGCGAAACGGAACAGCCGCACG | |
| B-F210A-fw | CGGCTTTCACGCATCAGC | Mutational screen |
| B-F210A-rv | GGCGGTTCTGGTGTCCTCGCG | |

| Primer name | Sequence (5’ -> 3’) | Purpose |
|---|---|---|
| C-F200A-fw | CGTTTCGACCGCATCGTCG | Mutational screen |
| C-F200A-rv | GGCCAGCCGCACGGCCTC | |
| B-R46A-R50A-fw | GACAAGAACGAGTTGCGCG | Mutational screen |
| B-R46A-R50A-rv | CGCCTCGAACATCGCCGAGAACGCCTCGTCAC | |
