# Supplementary material for: Structure and functional implications of WYL domain-containing bacterial DNA damage response regulator PafBC
Source: Nat Commun. 2019 Oct 11;10:4653. doi: 10.1038/s41467-019-12567-x (PMC6789036; doi:10.1038/s41467-019-12567-x)
Supplement: Supplementary file 3 — Description of Additional Supplementary Files [file 41467_2019_12567_MOESM3_ESM.pdf]

Description of Additional Supplementary Files

File name: Supplementary Data 1

Description: List of WYL domain-containing proteins in the superkingdom Bacteria. Letter codes in the "class" column correspond to domain architecture classes in Figure 6 and Supplementary Figure 11.

File name: Supplementary Data 2

Description: List of WYL domain-containing proteins in the superkingdoms Eukaryota, Archaea, and Viruses.
