# Supplementary material for: Structure and functional implications of WYL domain-containing bacterial DNA damage response regulator PafBC
Source: Nat Commun. 2019 Oct 11;10:4653. doi: 10.1038/s41467-019-12567-x (PMC6789036; doi:10.1038/s41467-019-12567-x)
Supplement: Supplementary file 6 — Reporting Summary [file 41467_2019_12567_MOESM6_ESM.pdf]

nature research

Corresponding author(s): Eilika Weber-Ban

Last updated by author(s): Sep 11, 2019

Reporting Summary

Nature Research wishes to improve the reproducibility of the work that we publish. This form provides structure for consistency and transparency in reporting. For further information on Nature Research policies, see Authors & Referees and the Editorial Policy Checklist .

Statistics

For all statistical analyses, confirm that the following items are present in the figure legend, table legend, main text, or Methods section.

n/a	Confirmed	
☐	☒	The exact sample size (n) for each experimental group/condition, given as a discrete number and unit of measurement
☐	☒	A statement on whether measurements were taken from distinct samples or whether the same sample was measured repeatedly
☒	☐	The statistical test(s) used AND whether they are one- or two-sided<br>*Only common tests should be described solely by name; describe more complex techniques in the Methods section.*
☒	☐	A description of all covariates tested
☒	☐	A description of any assumptions or corrections, such as tests of normality and adjustment for multiple comparisons
☒	☐	A full description of the statistical parameters including central tendency (e.g. means) or other basic estimates (e.g. regression coefficient) AND variation (e.g. standard deviation) or associated estimates of uncertainty (e.g. confidence intervals)
☒	☐	For null hypothesis testing, the test statistic (e.g. F, t, r) with confidence intervals, effect sizes, degrees of freedom and P value noted<br>*Give P values as exact values whenever suitable.*
☒	☐	For Bayesian analysis, information on the choice of priors and Markov chain Monte Carlo settings
☒	☐	For hierarchical and complex designs, identification of the appropriate level for tests and full reporting of outcomes
☒	☐	Estimates of effect sizes (e.g. Cohen's d, Pearson's r), indicating how they were calculated

Our web collection on statistics for biologists contains articles on many of the points above.

Software and code

Policy information about availability of computer code

Data collection	Swiss Light Source (SLS, Paul-Scherrer-Institut, Villigen, Switzerland) in-house software for beamline operation
Data analysis	HMMER v3.2.1, CD-HIT v4.6.8, ClustalO v1.2.4, Jalview v2.10.5, UCSF Chimera v1.12 build 41623, AL2CO, XDS (version Jan 26, 2018 BUILT=20180808), AIMLESS v0.7.3, SHELX, Phenix v1.14, Coot v0.8.9.2, GraphPad Prism v7.03

For manuscripts utilizing custom algorithms or software that are central to the research but not yet described in published literature, software must be made available to editors/reviewers. We strongly encourage code deposition in a community repository (e.g. GitHub). See the Nature Research guidelines for submitting code & software for further information.

Data

Policy information about availability of data

All manuscripts must include a data availability statement. This statement should provide the following information, where applicable:

- Accession codes, unique identifiers, or web links for publicly available datasets
- A list of figures that have associated raw data
- A description of any restrictions on data availability

Structural data are deposited in the protein data bank (PDB ID 6SJ9). Other datasets are either contained in the main manuscript or provided as supplementary or source data files. Other data are available from the corresponding author upon reasonable request.

Field-specific reporting

Please select the one below that is the best fit for your research. If you are not sure, read the appropriate sections before making your selection.

Life sciences study design

All studies must disclose on these points even when the disclosure is negative.

Sample size	Sample size per experiment and per strain was one, because multiple repeats of the experiment were carried out (see also replication).
Data exclusions	No data were excluded for the mutational screen.<br>Data exclusion for X-ray analysis was based on CC1/2 > 0.7
Replication	Experiments were repeated individually three or more times (i.e. in case of the mutational screen starting from transformation of bacteria with the protein variants). Results could be reproduced successfully each time.
Randomization	Experiments were repeated individually.
Blinding	Blinding is not applicable. Investigators need to able to identify the bacterial strains in the culture flasks at any given time due to safety regulations.

Reporting for specific materials, systems and methods

We require information from authors about some types of materials, experimental systems and methods used in many studies. Here, indicate whether each material, system or method listed is relevant to your study. If you are not sure if a list item applies to your research, read the appropriate section before selecting a response.

Materials & experimental systems

n/a	Involved in the study
☐	☒ Antibodies
☒	☐ Eukaryotic cell lines
☒	☐ Palaeontology
☒	☐ Animals and other organisms
☒	☐ Human research participants
☒	☐ Clinical data

Methods

n/a	Involved in the study
☒	☐ ChIP-seq
☒	☐ Flow cytometry
☒	☐ MRI-based neuroimaging

Antibodies

Antibodies used

anti-PafBC, anti-RpoB, anti-RecA

Validation

anti-PafBC - Fudrini Olivencia et al. 2017, Scientific Reports 7:13987, doi: 10.1038/s41598-017-14410-z

anti-RecA - MBL International, clone ARM414, monoclonal mouse antibody raised against recombinant E.coli RecA protein, antibody profile available online (https://www.acris-antikoerper.de/antibodies/primary-antibodies/recombinase-a-reca-am26666af-n.htm, 09.09.2019). References (as listed by manufacturer):
Mori, M., et al., Blood 94, 2744-2753 (1999)
Ikeda, M., et al., J. Biol. Chem. 267, 6291-6296 (1992)
Shibata, T., et al., Biochemin. 73, 209-217 (1991)
Ikeda, M., et al., J. Biol. Chem. 265, 8957-8965 (1990)
Ikeda, M., et al., J. Biol.Chem. 265, 8948-8956 (1990)
Makino, O., et al., J. Biol. Chem. 260, 15402-15405 (1985)
Shibata, T., et al., Methods Enzymol. 100, 197-209 (1983)

anti-RpoB - BioLegend, clone 8RB13, monoclonal mouse antibody raised against recombinant RNA polymerase subunit beta (RpoB), antibody profile available online (https://www.biolegend.com/en-us/products/anti-e-coli-rna-polymerase-beta-antibody-10494). Application references (as listed by manufacturer):
1. Bergendahl V, et al. 2003. Protein Expr. Purif. 31:155.
2. Burgess RR and Thompson NE. 2002. Curr. Opin. Biotechnol. 13:304.
3. Stalder, ES. et al. 2011. Protein Expr. Purif. 77(1):26-33. (Epitope, ELISA, IP, WB)
